# Healthcare Professionals’ Perceptions of AI-Assisted Clinical Decision-Making in Jordan: A Qualitative Study of Trust, Accountability, System Readiness, and Professional Practice

**DOI:** 10.3390/healthcare14121724

**Published:** 2026-06-15

**Authors:** Mohammad Abu Assab, Fares Al Bahar, Wael Abu Dayyih, Buthaina Mohammad Alazazmeh, Sewar W. Assaf, Anas Abed, Hayam A. Alrasheed, Zainab Zakaraya

**Affiliations:** 1 Clinical Pharmacy Department, Faculty of Pharmacy, Zarqa University, Zarqa 13132, Jordan; mabuassab@zu.edu.jo (M.A.A.); falbahar@zu.edu.jo (F.A.B.); 2 Faculty of Pharmacy, Mutah University, Al Karak 61710, Jordan; wabudayyih@mutah.edu.jo (W.A.D.); buthainamohammad999@gmail.com (B.M.A.); assafsewar01@yahoo.com (S.W.A.); 3 Department of Biopharmaceutics and Clinical Pharmacy, Faculty of Pharmacy, Al-Ahliyya Amman University, Amman 19328, Jordan; a.abed@ammanu.edu.jo; 4 Department of Pharmacy Practice, College of Pharmacy, Princess Nourah Bint Abdulrahman University, P.O. Box 84428, Riyadh 11671, Saudi Arabia; haalrasheed@pnu.edu.sa

**Keywords:** artificial intelligence, clinical decision support, qualitative research, healthcare professionals, Jordan, trust, accountability, health-system readiness, medication safety, LMIC

## Abstract

**Highlights:**

**What are the main findings?**

- Healthcare professionals in Jordan expressed cautious but constructive views toward AI-assisted clinical decision-making.
- Trust in AI was conditional and depended on local validation, transparency, institutional endorsement, and clear accountability.
- Participants supported AI as an adjunct to clinical judgement, particularly for screening, risk flagging, and information support.
- Medication-related safety emerged as a cross-cutting concern, particularly where AI recommendations could influence dosing, drug interactions, polypharmacy, or therapeutic monitoring.
- Participants linked safe AI implementation to governance, ethical data practices, workforce training, local validation, and equitable digital infrastructure.

**What are the implications of the main findings?**

- AI implementation in Jordanian healthcare should be approached as supervised clinical decision support rather than autonomous decision-making. Participants’ accounts suggest the need for local validation, clear accountability procedures, ethical data governance, and institutional policies defining how AI outputs are used, documented, challenged, or overridden.
- Interprofessional AI training is needed to help healthcare professionals critically appraise AI outputs, recognise limitations, and avoid over-reliance. Where AI tools influence medication-related decisions, medication-safety expertise, including pharmacist input where appropriate, should be considered.

**Abstract:**

Background/Objectives: Artificial intelligence (AI) is increasingly used in clinical decision-support systems, yet its adoption in low- and middle-income countries, including Jordan, remains limited and underexplored. Understanding how healthcare professionals perceive AI-assisted clinical decision-making is essential for safe and contextually appropriate implementation. This study explored healthcare professionals’ perceptions of AI-assisted clinical decision-making in Jordan, with particular attention to trust, accuracy, accountability, professional judgement, digital literacy, and health-system readiness. Medication-related safety and prescribing concerns were examined as secondary cross-cutting issues where they emerged from participants’ accounts. Methods: A qualitative study was conducted using semi-structured, in-depth interviews with 22 purposively sampled healthcare professionals from public, private, and university-affiliated healthcare institutions in Amman, Irbid, and Zarqa. Participants included physicians, nurses, pharmacists, and allied health professionals with varied specialties and levels of seniority. Data were analysed using Braun and Clarke’s reflexive thematic analysis. Member checking, peer debriefing, reflexive memos, and audit trails were used to enhance trustworthiness, and reporting followed the Consolidated Criteria for Reporting Qualitative Research (COREQ). Results: Eight overarching themes were identified: conditional trust in AI-assisted clinical decision-making; concerns regarding accuracy and confident algorithmic errors; accountability and professional responsibility; AI as an adjunct rather than a substitute for clinical judgement; the influence of experience, specialty, and digital literacy on AI acceptance; Jordanian health-system readiness; privacy, confidentiality, and algorithmic bias; and training requirements for safe AI use. Medication-related safety emerged as a cross-cutting concern, particularly in relation to dosing, polypharmacy, drug–drug and drug–herb interactions, and the risk of over-reliance on AI-generated recommendations. Conclusions: Healthcare professionals in Jordan expressed cautious but constructive views toward AI-assisted clinical decision-making. AI was perceived as potentially useful when used to support, rather than replace, professional judgement. Participants’ accounts suggest that safe implementation depends on local validation, clear accountability frameworks, ethical data governance, interprofessional training, and careful consideration of medication-safety expertise where AI tools influence prescribing or therapeutic decisions. These findings highlight the importance of context-sensitive AI governance strategies that support trustworthy, accountable, and professionally supervised AI adoption in healthcare.

## 1. Introduction

The integration of artificial intelligence (AI) into healthcare represents one of the most consequential technological shifts in contemporary clinical practice. AI-assisted systems are increasingly being applied across diagnostic imaging, clinical documentation, predictive analytics, clinical decision support, and population-health risk stratification, with the potential to improve efficiency, reduce variability in care, and support evidence-informed decision-making [1,2,3]. In clinical settings, AI may assist healthcare professionals by identifying patterns in complex datasets, generating decision-support recommendations, and supporting tasks that would otherwise place substantial cognitive or administrative burden on clinicians. However, alongside these potential benefits, AI introduces important concerns regarding algorithmic opacity, data bias, clinical validity, professional autonomy, accountability, privacy, and the risk of over-reliance on automated recommendations [4,5].

Importantly, the safe use of AI in healthcare is increasingly understood as a lifecycle process rather than a single act of technological adoption. AI-enabled clinical systems require careful development, external validation, workflow integration, user training, monitoring after deployment, and mechanisms for detecting performance drift, bias, and unintended consequences [6,7]. This lifecycle perspective is particularly relevant in clinical decision-support contexts, where an AI output may influence diagnosis, triage, prescribing, or monitoring decisions. Therefore, questions of trust, accountability, and safety are not limited to whether clinicians are willing to use AI, but extend to how AI systems are selected, validated, governed, updated, monitored, and embedded within clinical workflows [8,9].

Despite the rapid growth of AI research in healthcare, much of the evidence base originates from high-income settings, particularly North America, Western Europe, and parts of East Asia, where digital infrastructure, health data ecosystems, regulatory capacity, and implementation resources differ substantially from those of low- and middle-income countries (LMICs) [10,11]. In LMIC contexts, AI implementation cannot be understood solely as a technical process; it is shaped by health-system readiness, regulatory maturity, workforce preparedness, infrastructure, local clinical validation, and the distribution of professional responsibilities within care teams. Jordan, a middle-income country in the Eastern Mediterranean region, provides an important context for examining these dynamics because its healthcare system combines increasing interest in digital transformation with persistent challenges related to interoperability, resource variation between sectors, workforce pressures, and evolving governance structures.

Conceptually, AI-assisted clinical decision-making can be understood through a sociotechnical lens, in which technology adoption is shaped by the interaction between digital tools, professional roles, institutional policies, clinical workflows, and patient-safety norms [12,13]. This perspective is useful because AI does not enter clinical practice as a neutral technical object; rather, its meaning and acceptability are negotiated by healthcare professionals within existing structures of authority, responsibility, trust, and resource availability. In addition, theories of trust in automation and institutional trust suggest that clinicians’ willingness to rely on AI is likely to depend not only on perceived technical accuracy, but also on transparency, explainability, organisational endorsement, legal accountability, and confidence in the systems responsible for implementation [6,7].

Recent Jordanian evidence has begun to examine healthcare professionals’ perceptions of AI use in clinical practice. For instance Dalky et al. (2025) conducted a cross-sectional survey of 605 healthcare professionals across multiple hospital types in Jordan and reported generally optimistic perceptions of AI integration, alongside moderate perceived barriers and risks related to preparedness, training, safety, trust, regulation, and responsibility [14]. Similarly, a physician-focused Jordanian cross-sectional survey examined physicians’ willingness to integrate AI into clinical practice and highlighted ethical and practical considerations surrounding AI adoption among physicians [15]. Another questionnaire-based Jordanian study reported receptive attitudes among healthcare professionals toward AI adoption, while identifying concerns regarding job stability and the potential replacement of professional roles [16]. Research among Pharm-D students in Jordan has also examined perceptions, concerns, and practices related to ChatGPT in clinical training [17]. Organisational research from Jordan has further linked AI and big-data analytics to healthcare outcomes, supporting the relevance of institutional capability and implementation context [18]. These studies are valuable because they demonstrate that AI is increasingly being considered within Jordanian clinical and organizational contexts. However, they remain primarily survey-based and therefore provide limited insight into how healthcare professionals interpret AI-generated recommendations when clinical uncertainty, patient safety, institutional accountability, professional hierarchy, and medication-related risk are involved. Survey designs can identify levels of acceptance or concern, but they are less able to explain how clinicians decide whether an AI recommendation should be trusted, questioned, overridden, documented, or escalated. This qualitative gap is particularly important in Jordan and similar LMIC settings, where AI implementation is shaped not only by individual attitudes but also by infrastructure, regulation, local validation capacity, interprofessional workflow, and institutional readiness.

Medication-related decision-making represents one clinically important area in which AI-assisted decision support may carry direct patient-safety implications. Prescribing decisions, drug therapy optimization, medication reconciliation, polypharmacy management, dose adjustment, and the identification of clinically meaningful drug–drug or drug–herb interactions require the integration of clinical guidelines, patient-specific factors, comorbidities, organ function, concurrent medication use, therapeutic goals, adherence behavior, and feasibility of monitoring. AI-based clinical decision support systems may assist with structured medication-related tasks, but their reliability in complex prescribing scenarios depends on the quality of training data, local validation, workflow integration, and the clinician’s ability to critically evaluate the output [19,20]. In Jordan and similar LMIC contexts, these issues maybe influenced by local prescribing practices, antimicrobial resistance patterns, medication availability, polypharmacy burden, and the use of complementary or herbal products.

Healthcare professionals constitute the decisive human interface through which AI tools either become safely integrated into practice or remain underused, misused, or resisted. Their perceptions of trust, accuracy, accountability, clinical usefulness, and professional responsibility are therefore central to understanding AI adoption [21,22]. However, measuring attitudes or willingness alone is insufficient. Healthcare professionals do not simply “accept” or “reject” AI in the abstract; rather, they evaluate AI outputs in relation to patient risk, institutional endorsement, professional liability, clinical uncertainty, and the perceived limits of algorithmic reasoning. By focusing on how healthcare professionals reason through AI-related trust, accountability, safety, and system-readiness concerns, this study provides a more interpretive account of AI adoption than can be obtained from attitude measurement alone. Its contribution lies in explaining how AI acceptance is constructed conditionally through the interaction of technical confidence, professional responsibility, institutional legitimacy, and contextual readiness.

This study therefore addresses a specific empirical gap by conducting an interview-based qualitative exploration of healthcare professionals’ perceptions of AI-assisted clinical decision-making in Jordan. Rather than measuring general acceptance, attitudes, or willingness to use AI, the study examines how healthcare professionals interpret, question, and negotiate AI-generated recommendations in relation clinical judgment, patient safety, professional accountability, and system readiness. Medication-related safety was explored as a secondary cross-cutting issue because AI-supported recommendations may influence prescribing, dosing, interaction detection, and therapeutic monitoring. Guided by reflexive thematic analysis, this study seeks to explain not merely whether healthcare professionals are receptive to AI, but under what conditions they consider AI-assisted clinical decision-making to be trustworthy, safe, and professionally accountable.

## 2. Materials and Methods

This section is reported in accordance with the 32-item Consolidated Criteria for Reporting Qualitative Research (COREQ) checklist, which covers three domains: research team and reflexivity, study design, and data analysis and reporting. The completed COREQ checklist is provided in Supplementary File S1 [23].

### 2.1. Research Team and Reflexivity

#### 2.1.1. Personal Characteristics

The interviews were conducted by the principal investigator (PI) or a trained research assistant. The PI is an academic researcher with doctoral-level training and experience in qualitative health research, clinical pharmacy, pharmacotherapy, and health informatics. The wider research team included researchers with expertise in clinical pharmacy, medical ethics, health informatics, and health policy. The research team included both male and female members. All interviewers were trained in qualitative interviewing and were familiar with the study protocol before data collection commenced.

No member of the research team held a dual clinical-supervisory relationship with any participant. To mitigate potential hierarchical influence on the data, interviews were conducted by the PI or by a trained research assistant who was not affiliated with the participants’ employing institutions.

#### 2.1.2. Relationship with Participants

Prior to data collection, no pre-existing relationships existed between the research team and participants. Contact was first established through institutional gatekeepers at each study site. Participants were informed of the researchers’ academic affiliation, the study’s aims, and the voluntary, confidential nature of participation prior to consenting. Interviewers disclosed their professional background to participants at the outset of each interview, consistent with reflexive qualitative practice.

#### 2.1.3. Researcher Reflexivity

The research team maintained reflective journals throughout data collection and analysis. Reflexive memos documented positionality, emerging assumptions, and analytic decisions. Team members acknowledged pre-existing views about AI’s potential in healthcare, including in pharmacotherapy and clinical decision support, and committed to bracketing these through regular debriefing sessions and peer review of coded data. The PI’s professional background in clinical pharmacy warranted explicit reflexive attention, particularly because medication safety and prescribing-risk themes were prominent in the dataset. To avoid overemphasising any single professional group, the research team reviewed quotation distribution across participants and disciplines during the final analytic phase. Representative quotations were selected to reflect both thematic salience and professional diversity.

Because several members of the research team had backgrounds in clinical pharmacy, pharmacotherapy, health in-formatics, and healthcare education, particular attention was paid to the possibility that medication-safety and pre-scribing-related issues could be over-interpreted. During analysis meetings, the team therefore actively reviewed whether pharmacotherapy-related interpretations were supported by multiple participants and whether they were being treated as cross-cutting concerns rather than as pharmacist-specific conclusions. Reflexive memos were used to document these analytic decisions and to distinguish participant-generated meanings from the researchers’ disciplinary assumptions.

### 2.2. Study Design

#### 2.2.1. Methodological Orientation

A qualitative research design was adopted, guided by a constructivist epistemological orientation and analysed using reflexive thematic analysis as developed by Braun and Clarke (2006, 2021) [24]. Reflexive thematic analysis was selected for its theoretical flexibility, methodological transparency, and suitability for identifying patterned meanings across a diverse dataset while acknowledging the researcher’s active interpretive role in theme construction.

The constructivist orientation shaped the analysis by treating participants’ accounts not as objective measurements of AI readiness, but as situated interpretations produced within specific professional, institutional, and health-system contexts. Accordingly, themes were understood as interpretive patterns constructed through engagement between participants’ accounts, researcher reflexivity, and the study context. This orientation was appropriate because the study aimed to understand how healthcare professionals make meaning of AI-assisted decision-making, rather than to quantify the prevalence of predefined attitudes.

#### 2.2.2. Participant Selection

Purposive sampling was employed to ensure maximum variation across professional discipline, clinical specialty, seniority, gender, sector of employment (public, private, university-affiliated), and geographic location within Jordan. Eligibility criteria required participants to be (a) registered and currently practising healthcare professionals in Jordan; (b) employed in a clinical role involving direct patient care; and (c) having some degree of awareness of or exposure to digital health tools or clinical decision-support systems, whether through direct use, institutional pilot programmes, or professional development activities. Because medication-related decision-making was a secondary area of interest, pharmacists and clinicians with regular prescribing or medication-review responsibilities were purposively included. However, the study was not designed as a pharmacist-specific investigation, and pharmacists represented a small subgroup of the final sample.

Exclusion criteria included healthcare administrators without direct clinical responsibilities and individuals currently enrolled as students. Recruitment proceeded until thematic saturation was reached, the point at which no substantively new themes or concepts emerged from successive interviews. A final sample of 22 participants was achieved.

#### 2.2.3. Setting

Data were collected between November 2025 and February 2026 across eight healthcare institutions in three Jordanian governorates: Amman, Irbid, and Zarqa. Sites included public, private, and university-affiliated hospitals, primary healthcare centres, and one rehabilitation centre. This multi-site approach was intentional in capturing variation in technological resources, institutional culture, and patient demographics across the Jordanian health system.

#### 2.2.4. Data Collection

Individual, in-depth semi-structured interviews were the primary data collection method. The interview guide was developed iteratively by the research team using established methodological guidance for semi-structured interview design and qualitative health research, alongside a focused review of literature on AI in healthcare, clinical decision support, trust, accountability, and medication safety [25,26]. To ensure content relevance and clarity, the guide was reviewed and pilot-tested with three healthcare professionals not included in the main sample: one physician with experience in digital health/clinical decision support, one clinical pharmacist, and one nurse with experience in hospital-based patient care. Feedback focused on question clarity, sequencing, terminology, sensitivity, and whether the guide adequately captured AI-related clinical reasoning, prescribing safety, accountability, and system-readiness issues. Minor refinements were made before formal data collection. The complete interview guide is provided as Supplementary File S2.

The interview guide included core open-ended questions on participants’ awareness of AI in healthcare, perceived clinical usefulness, trust, accuracy, accountability, professional responsibility, data privacy, health-system readiness, and training needs. All participants were asked the same core questions to ensure consistency across professional groups. Flexible probes were then used to explore issues raised by participants in greater depth. For example, participants who discussed medication-related decisions were asked follow-up questions about prescribing, dosing, drug interactions, medication reconciliation, and pharmacist involvement. Participants who discussed institutional implementation were asked additional probes about governance, documentation, escalation pathways, and organisational responsibility. In this way, the interview guide sensitised the interviews to relevant domains, but the depth and direction of each discussion were shaped by participants’ own accounts.

Participants were also asked to clarify the basis of their views, including whether they had directly used AI-enabled tools, used conventional digital clinical decision-support systems, attended AI-related training or institutional discussions, or were reflecting on anticipated future applications of AI in healthcare. This distinction was recorded in field notes and considered during analysis, because perceptions based on direct experience may differ from perceptions based on indirect exposure or hypothetical expectations.

All interviews were conducted in participants’ preferred language, Arabic (*n* = 17) or English (*n* = 5), and took place as face to face interviews in private rooms to ensure confidentiality and minimise interruptions. Interviews lasted between 45 and 60 min. With participants’ written informed consent, all interviews were audio-recorded and subsequently transcribed verbatim. Arabic-language transcripts were translated into English by a bilingual researcher with clinical and methodological expertise. Back-translation was conducted for five transcripts, corresponding to approximately 20% of the dataset, by a second independent bilingual researcher to verify semantic accuracy. The transcripts were selected using a computer-generated random sequence from the Arabic-language interviews and checked to ensure variation by professional role and institution type where possible. Discrepancies were resolved by discussion between bilingual research-ers and the wider research team, with priority given to semantic and contextual equivalence rather than literal word-for-word translation. Quotations appearing in the manuscript reflect the translated text as verified through this process; where the polished register of English quotations reflects the original Arabic register rather than editorial smoothing, this is a feature of the participants’ own formal professional speech. No quotations were substantively edited beyond translation.

To preserve authenticity, translated quotations were checked against the original Arabic transcripts before inclusion in the manuscript. Quotations were edited only minimally for grammar, readability, and removal of identifiable details; no substantive meaning was added, removed, or rhetorically enhanced. Where participants used formal or meta-phorical Arabic expressions, the translation aimed to preserve the intended meaning while avoiding unnecessary stylistic embellishment. During revision, representative quotations were reviewed again to ensure that they remained faithful to the original transcript and did not overstate the rhetorical polish of participants’ accounts.

Field notes capturing non-verbal communication, environmental context, and reflexive observations were recorded immediately following each interview.

#### 2.2.5. Sample Size

The target sample was guided by the principle of informational redundancy [27]. Data collection and analysis proceeded iteratively, and the research team reviewed emerging codes and candidate themes after every five interviews. A saturation log was maintained to document whether each interview generated new codes, extended existing codes, or contributed only confirmatory examples to already-developed themes. By the twen-tieth interview, no substantively new codes or candidate themes were identified at the level of the overall dataset, and the existing thematic structure was sufficiently populated across the sample as a whole. Two additional interviews were conducted to confirm informational redun-dancy and to ensure that no major new conceptual categories emerged. Importantly, saturation was assessed across the overall dataset rather than separately within each professional subgroup. Given the small number of pharmacists and allied health professionals, we do not claim discipline-specific saturation for these groups. Instead, the final sample was considered sufficient for identifying cross-professional themes re-lated to AI-assisted decision-making, while subgroup-specific findings, particularly those related to pharmacists and allied health professionals, are interpreted cautiously. A final sample of 22 participants was therefore considered appropriate for the study aim and analytic approach. This sample size is consistent with established norms for reflexive thematic analysis studies in health professions research [28].

#### 2.2.6. Non-Participation

Of 31 clinicians initially approached, 22 agreed to participate. Five declined due to time constraints, and four did not respond to follow-up communications. Non-participants did not differ systematically from participants in terms of professional discipline or institutional affiliation as far as could be determined from available information.

### 2.3. Data Analysis and Trustworthiness

#### 2.3.1. Data Analysis

Reflexive thematic analysis following Braun and Clarke’s (2006; 2021) [24] six-phase framework was employed: (1) familiarisation through repeated reading of transcripts and field notes; (2) systematic generation of initial codes applied to the entire dataset; (3) searching for themes by clustering codes into candidate thematic groupings; (4) reviewing themes for coherence, internal homogeneity, and external heterogeneity; (5) defining and naming themes with precision; and (6) producing the report.

Coding was conducted at both semantic and latent levels. Semantic codes captured explicit statements, such as concerns about incorrect AI recommendations, unclear liability, or lack of training. Latent coding was used to interpret underlying patterns, such as conditional trust, institutional dependence, perceived loss of professional control, and concern about automation bias. Initial codes were then compared across transcripts and grouped into broader candidate themes. For example, codes related to local validation, institutional approval, explainability, and repeated performance were clustered into the candidate theme of conditional trust. Codes related to unclear legal responsibility, professional liability, documentation, and escalation were clustered into the accountability theme. Medication-related codes, including dosing, polypharmacy, renal adjustment, drug-drug interactions, and drug-herb interactions, were reviewed separately to assess whether they reflected a distinct pharmacist-specific theme or a broader patient-safety concern. Because these codes were raised by pharmacists and by several non-pharmacist participants in relation to trust, accuracy, accountability, and human oversight, they were interpreted as a cross-cutting concern rather than as a stand-alone pharmacotherapy theme. The two coders met regularly to compare coding decisions, discuss divergent interpretations, and refine the developing codebook. Disagreements were not resolved through statistical inter-rater reliability testing, which is not required in reflexive thematic analysis, but through reflexive discussion, return to the source transcripts, and review by the wider research team. Analytic decisions, theme revisions, and reasons for merging or separating themes were documented in an audit trail.

Analysis was conducted using NVivo 14 (QSR International, Melbourne, Australia). Initial coding was undertaken independently by two team members, with coding consistency verified through regular comparison meetings. Divergent interpretations were resolved through discussion and recourse to source transcripts. A reflexive audit trail documented all analytic decisions, revisions, and rationale for theme consolidation.

#### 2.3.2. Derivation of Themes

Themes were developed primarily inductively from participants’ accounts rather than imposed from a pre-existing theoretical framework. However, the interview guide included sensitising domains, such as trust, accountability, privacy, training, system readiness, and medication-related safety, because these areas were identified as relevant in the literature and in the study objectives. These domains informed the interviews but did not determine the final thematic structure. During analysis, the research team distinguished between issues that were directly prompted by the guide and issues that participants raised spontaneously or elaborated beyond the prompts. Accountability, local validation, training needs, and system readiness were discussed across inter-views both in response to prompts and spontaneously as participants reflected on real or anticipated AI use. Medication-related safety was explored through follow-up probes when participants discussed pre-scribing or therapeutic decision-making and was therefore interpreted as a cross-cutting concern rather than as a primary theme.

A supplementary coding tree illustrating the progression from selected initial codes to candidate themes and final themes is provided in Supplementary File S3.

Although the analysis was primarily inductive, the final interpretation was informed by relevant literature on professional trust, clinical decision-making, accountability, medication safety, and digital health implementation. These concepts were used to support interpretation in the Discussion rather than to predetermine the coding process. Representative quotations were selected to illustrate the analytic meaning of each theme while reflecting variation across professional roles, seniority levels, and institutional settings.

#### 2.3.3. Participant Feedback

Preliminary theme summaries were shared with a purposive subsample of eight participants selected to reflect variation in professional role, seniority, sector, and geographic location. Participants were invited to comment on whether the preliminary interpretations resonated with their experiences and whether any important perspectives appeared to be missing. Feedback was used to refine wording, clarify interpretation, and ensure that the findings remained meaningfully connected to participants’ accounts. No participant requested withdrawal of their data or raised concerns about the overall thematic interpretation.

### 2.4. Ethical Considerations

Ethical approval was granted by the Institutional Review Board of Al-Ahliyya Amman University (IRB No. AAU/1/9/2024-2025). All participants provided written informed consent prior to participation. Participants were assured of voluntary participation, the right to withdraw at any time without consequence, and the confidentiality of their data. All transcripts were de-identified prior to analysis, and pseudonyms are used in all reporting. Audio recordings were stored on password-protected, encrypted servers accessible only to the core research team and deleted following verification of transcript accuracy. Participant and institutional characteristics are summarized in Table 1.

## 3. Results

**Figure 1:**
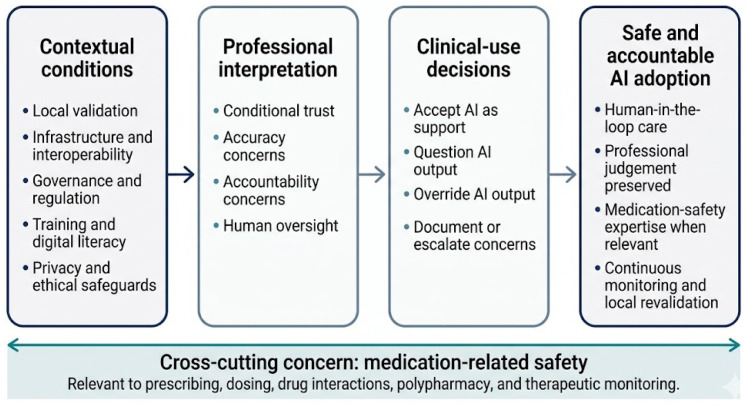
Conceptual model of healthcare professionals’ perceptions of AI-assisted clinical decision-making in Jordan. The model shows how healthcare professionals’ acceptance of AI-assisted clinical decision-making was shaped by the interaction between local validation, institutional governance, accountability clarity, professional judgement, training, and system readiness. AI adoption was perceived as safest when AI functioned as a human-supervised support tool embedded within accountable clinical workflows.

Analysis of 22 in-depth interviews generated eight overarching themes describing how healthcare professionals perceived AI-assisted clinical decision-making in Jordan. The themes were analytically distinct but interrelated. Conditional trust was shaped by perceived accuracy, local validation, institutional endorsement, and accountability clarity. Accountability concerns were closely linked to system readiness, because participants were reluctant to rely on AI tools in the absence of clear governance, documentation procedures, and legal responsibility. Training and digital literacy influenced whether participants felt able to critically evaluate AI outputs, while medication-related safety emerged as a cross-cutting concern when participants discussed prescribing, dosing, drug interactions, and therapeutic monitoring. Table 2 provides a summary of the eight themes, their analytical focus, and representative quotations, while Figure 1 illustrates the conceptual relationships among the themes and how they contribute to safe and accountable AI adoption.

Although the eight themes were shared across professional groups, the emphasis differed by role. Physicians tended to frame AI in relation to diagnostic responsibility, clinical autonomy, and medico-legal liability. Pharmacists focused more strongly on medication-related safety, including dosing, interactions, and polypharmacy. Nurses frequently discussed institutional approval, bedside workflow, and escalation procedures, while allied health professionals emphasised training access, role clarity, and digital literacy. These differences are summarised in Table 3.

Participants’ accounts reflected varying levels of exposure to AI and digital decision-support systems. Some participants described direct or indirect experience with AI-enabled or algorithm-supported tools, particularly in imaging, electronic health records, clinical alerts, or institutional discussions about digital transformation. Others reflected mainly on anticipated future applications of AI in clinical practice. Therefore, the findings should be interpreted as perceptions of AI-assisted decision-making and readiness rather than as observations of routine AI use across Jordanian healthcare settings.

### 3.1. Theme 1: Conditional Trust in AI-Assisted Clinical Decision-Making

Trust was the dominant lens through which participants evaluated AI-assisted clinical decision-making. However, trust was not expressed as unconditional acceptance. Participants described AI as potentially useful only when its outputs were locally vali-dated, transparent, clinically explainable, and institutionally endorsed. However, trust was not expressed as categorical or unconditional. Instead, it was consistently framed as provisional, context-specific, and dependent on perceived accuracy, institutional en-dorsement, transparency, and alignment with professional responsibilities. This condi-tional character of trust indicates that participants neither rejected AI outright nor accepted it uncritically.

Senior clinicians, in particular, articulated nuanced hierarchies of trust that distinguished between AI as a source of preliminary information and AI as a co-decision-maker. A senior cardiologist (P7) captured this distinction vividly:

*“I would not fully trust it immediately. It needs to prove that it works safely in our patients and in our hospital.”*

This metaphor of professional probation was echoed across multiple participants and encapsulates a relational model of trust in which AI must demonstrate consistent, verifiable, and contextually appropriate performance before clinicians will fully integrate its recommendations into their decision-making workflows. P15, a consultant neurologist with 18 years of practice, extended this reasoning to emphasise the conditional nature of AI validation:

*“Trust is not binary. I trust AI more for pattern recognition in imaging than I trust it to interpret why a patient is non-adherent to their medication. The moment it tries to do everything, I trust it less.”*

For junior clinicians, trust dynamics were qualitatively different. Several less-experienced participants expressed higher baseline enthusiasm for AI tools, which they framed as potentially compensatory for their own perceived gaps in clinical experience. P5, an emergency medicine physician in his second year of practice, noted:

*“When you are new, you second-guess yourself constantly. An AI that says ‘consider pulmonary embolism’ when you might have missed it—that is reassuring. It is like having a silent supervisor.”*

This divergence between junior and senior clinicians’ trust orientations is analytically significant. Whereas experienced practitioners constructed trust as a professional judgement earned through observed performance, junior practitioners were more inclined to experience AI as a form of cognitive scaffolding-augmenting rather than replacing their developing clinical reasoning. This tension has direct implications for training and governance, as discussed in subsequent themes.

Nurses in the sample positioned trust differently again, tending to frame AI acceptance in terms of institutional legitimacy. P6, a senior oncology nurse, reflected:

*“I would trust it if the hospital says it is verified and safe. My trust in the tool depends on whether the people responsible for this hospital trust it. I cannot independently evaluate an algorithm.”*

This institutionally mediated trust, in which individual professional acceptance is delegated to or dependent on organisational endorsement, underscores the critical role of health system leadership in shaping frontline adoption. Participants across disciplines consistently returned to the importance of transparent, institution-level validation processes as a precondition for personal trust in AI tools.

### 3.2. Theme 2: Accuracy, Evidence, and the Risk of Clinical Misinformation

Concerns about AI accuracy were among the most consistently expressed reservations. Participants were not only concerned that AI might be wrong, but that it might be wrong in ways that appear confident, difficult to detect, or persuasive to less-experienced clinicians. Unlike trust, which was articulated in relational and institutional terms, ac-curacy concerns were framed in explicitly clinical and epistemic language—centered on error rates, evidence quality, generalizability, and the consequences of acting on faulty algorithmic outputs in high-stakes clinical environments.

Participants expressed concern not merely that AI might be occasionally inaccurate, but that the nature of AI error (confident, systematic, and potentially undetectable by less-experienced clinicians) represented a qualitatively distinctive clinical risk. P11, a consultant surgeon, elaborated:

*“When a junior doctor makes an error, it is usually detectable (in the history they missed, the examination they did not do, the uncertainty in their voice). The problem is that AI may give an answer with confidence. If the answer is wrong, a junior doctor may not feel able to question it.”*

This concern with confident miscalibration, AI systems presenting erroneous conclusions with high apparent certainty, was raised by 17 of 22 participants and represents a central axis of clinical scepticism. The danger was understood to be particularly acute in settings where less-experienced clinicians, under time pressure and cognitive load, might anchor inappropriately on AI recommendations without engaging in independent critical appraisal.

Participants also raised concerns about the evidential basis of AI systems deployed in Jordan. Several noted that AI models trained predominantly on Western clinical datasets might not adequately represent the epidemiological profile, comorbidity patterns, or treatment contexts characteristic of Jordanian and broader Middle Eastern patient populations. P21, an infectious disease specialist, articulated this directly:

*“Antimicrobial resistance patterns in Jordan are not the same as in the United States. If an AI is trained on American prescribing data and makes recommendations for our patients, we could be looking at inappropriate antibiotic guidance. That is not a small error—that is a patient safety catastrophe waiting to happen.”*

Pharmacists in the sample raised comparably structured concerns about medication dosing algorithms. P3 described a scenario in which an AI-based clinical decision support tool had recommended a standard adult dosage that did not account for the patient’s herbal medication use, a practice more prevalent in Jordanian clinical contexts than algorithms trained on Western datasets would typically capture:

*“Our patients use herbal treatments and do not always disclose them. They do not think it is medicine. An AI that does not ask, or does not know to ask, will give you a drug interaction risk it cannot even see.”*

This observation carries substantial pharmacotherapy implications: AI-driven prescribing recommendations that fail to account for undisclosed complementary medication use, a documented patient behaviour pattern in Arab clinical settings, risk producing clinically significant drug-herb interactions that are, by definition, invisible to the algorithm. P13, a hospital pharmacist, extended this concern to the domain of renally adjusted dosing, noting that AI tools validated on Western populations frequently misclassify the renal function categories of elderly Jordanian patients due to differences in body composition reference values, a failure mode with direct prescribing safety consequences. The risk is not merely one of commission, an inappropriate recommendation, but of false reassurance: a clean AI output interpreted as medication safety clearance when the system has fundamentally failed to capture the patient’s pharmacological risk profile.

The data further revealed a sophisticated understanding among participants of the distinction between AI as a diagnostic tool and AI as a decision tool. Many clinicians expressed greater comfort with AI in screening and pattern-detection roles, where the cost of a false positive could be absorbed through subsequent human clinical review, than in definitive decision-support contexts where an AI error might directly drive clinical action:

*“I am comfortable with AI flagging an abnormal ECG. I am deeply uncomfortable with AI telling me what drug to prescribe. Flagging creates a conversation. Prescribing closes it.”*

This distinction between AI as alerting versus AI as recommending maps onto a spectrum of perceived clinical risk and has particularly salient implications for the design of pharmacotherapy decision-support tools, where the transition from recommendation to prescription represents a legally and clinically irreversible act with direct patient safety consequences.

### 3.3. Theme 3: Accountability, Liability, and the Boundaries of Professional Responsibility

Responsibility for AI-assisted decisions emerged as one of the most legally and ethically charged issues. Participants were uncertain who would be accountable if an AI-influenced recommendation contributed to patient harm. Across the sample, there was near-universal consensus that the current absence of clear legal frameworks governing AI liability in Jordan created a professional and institutional vacuum that substantively undermined clinicians’ willingness to rely on AI recommendations in practice.

Participants consistently articulated what might be termed the liability paradox of AI-assisted decision-making: clinicians are expected to exercise professional judgement, yet the introduction of an AI recommendation creates a novel layer of evidentiary complexity in determining the causal chain of clinical decisions. P9, a consultant psychiatrist, framed this with precision:

*“If I follow the AI’s recommendation and the patient is harmed, am I liable? Probably yes—because I am the clinician of record. But what if the AI’s recommendation was wrong? Who is liable then? The hospital? The software company? There is no answer to that question in Jordanian law. And that absence makes every AI-assisted decision feel like standing on sand.”*

This metaphor of foundational legal instability was evocative of a broader anxiety shared across disciplines and seniority levels. For senior clinicians, the accountability concern was primarily expressed in terms of medico-legal exposure and the potential erosion of professional reputation in cases of adverse events attributed to AI-influenced decisions. For junior clinicians, the concern was more immediate: without clear guidance, AI recommendations became a source of cognitive and moral uncertainty rather than support.

P7 raised the particularly fraught scenario of divergence between AI recommendation and clinical judgement:

*“Imagine the AI says one thing and I decide another—and the patient has a bad outcome. If the outcome matches what the AI said, I will be asked why I did not follow it. If the AI was wrong, I am the professional, so I still bear the responsibility. It is not a level playing field.”*

Within the pharmacotherapy domain, this accountability paradox acquires additional clinical and legal specificity. Prescribing is a legally circumscribed act in Jordan, with clear statutory accountability attached to the prescriber of record. When an AI clinical decision support system generates a drug recommendation, whether for initial prescribing, dose adjustment, or drug substitution, and that recommendation is followed without adequate independent pharmacological verification, the resulting liability architecture is deeply ambiguous. P3, a senior clinical pharmacist, described this concern clearly:

*“If an AI tells a doctor to prescribe Drug A, and the doctor prescribes it, and the patient has a serious adverse drug reaction that a pharmacist reviewing the full medication history would have caught—who is accountable? The doctor who prescribed? The pharmacist who was not consulted? The AI that did not know what it did not know? This is not a hypothetical. It is a scenario that is happening right now, in hospitals without proper pharmacist integration.”*

This account foregrounds the structural risk of AI-mediated prescribing in contexts where the pharmacist’s role as an independent safety checkpoint is not systematically embedded in the clinical workflow, a particularly pressing concern in Jordanian public sector facilities where clinical pharmacy services remain underdeveloped relative to the complexity of the medication burden carried by hospitalised patients.

Nursing participants expressed accountability concerns that were specifically structured around professional hierarchy and communication norms. P2, an ICU nurse, described the difficulty of raising concerns about AI recommendations to medical colleagues:

*“If a doctor orders something based on what the AI said, and I think it is wrong, what do I say? ‘The AI might be wrong’? That is not how it works here. The accountability flows upward and stays there. Nurses carry risk but not authority.”*

This observation reveals an intersection between AI accountability discourse and pre-existing professional power structures in Jordanian healthcare—a dimension that is largely absent from Western-centric AI accountability frameworks but that is directly relevant to implementation contexts characterised by hierarchical clinical cultures. Participants across disciplines argued compellingly that AI governance frameworks in Jordan must explicitly address interprofessional accountability dynamics, rather than defaulting to individualised clinician responsibility models.

Several participants advocated for a shared or distributed accountability model in which AI developers, healthcare institutions, and individual clinicians each bear clearly delineated responsibilities with legal protection extended to clinicians who act in good faith on validated AI outputs. In the absence of such frameworks, many indicated they would err decisively toward conventional clinical practice, regardless of AI recommendations, as a professional self-protective strategy.

### 3.4. Theme 4: AI as an Adjunct to, Not a Substitute for, Clinical Judgment

Across professional groups, participants consistently emphasized that AI should support, not replace, human clinical judgement. This position was grounded less in technophobia than in concerns about patient context, professional responsibility, and the relational nature of clinical care. Participants from all professional backgrounds, spe-cialties, and levels of experience returned, often unprompted, to the categorical assertion that AI must function as a supplementary aid rather than as an autonomous or co-equal decision-maker.

This position was not framed as mere professional protectionism or technophobia; rather, it was grounded in sophisticated epistemological claims about the nature of clinical reasoning, the role of embodied professional knowledge, and the irreplaceable dimensions of the patient-clinician relationship. P15 articulated this with philosophical precision:

*“Clinical judgement is not just data processing. It is the integration of what the patient says, what they do not say, how they look when they say it, what I know from 20 years of seeing similar patients, and what my gut—shaped by all of that experience—tells me. No algorithm processes all of that. And it never will.”*

This view of clinical knowledge as experience-based, relational, and context-dependent was echoed across disciplines. Nurses, in particular, emphasised the affective and relational dimensions of clinical judgement that they regarded as inaccessible to algorithmic formalisation. P6 reflected:

*“When I assess a patient’s pain, I am not just reading a number on a scale. I am watching how they breathe, how they shift in the bed, whether their eyes are tracking me. AI may read a score, but we also look at the patient’s face, breathing, pain, and general condition.”*

Participants with backgrounds in clinical specialties that rely heavily on procedural skill and intraoperative decision-making such as surgery, anaesthesia, and obstetrics, were particularly emphatic that real-time clinical action involves a form of expertise that cannot be reduced to or directed by decision-support algorithms operating outside the temporal and sensory immediacy of the clinical encounter. P11 noted:

*“In surgery, your decision changes in seconds based on what you see and feel when you open. No AI is in the room with you. The moment of decision is mine alone.”*

Within the pharmacotherapy domain, the adjunct positioning of AI was articulated with particular nuance by pharmacist participants, who drew a clear operational distinction between AI as a screening tool and AI as a therapeutic decision instrument. P3 described the appropriate scope of AI in medication management as encompassing high-volume, rule-based tasks such as drug interaction flagging, formulary compliance checking, dose-range verification, while insisting that individualised pharmacotherapy decisions, such as drug selection in the context of complex comorbidities, renal or hepatic impairment, or polypharmacy, require the integrative reasoning of a trained clinical pharmacist that no current algorithm can substitute:

*“I welcome AI that tells me there is a potential interaction between two drugs in a complex patient’s list. I use that as a starting point. But the decision about what to do about it—whether to discontinue, substitute, adjust the dose, monitor more closely, or accept the risk given the patient’s priorities—that is pharmacist reasoning. That is not something I would ever delegate to a system that has not seen the patient, spoken to the family, or reviewed the full clinical trajectory.”*

This account of tiered AI utility, embracing algorithmic support for data-intensive screening while preserving professional sovereignty over therapeutic reasoning, represents the most clinically mature model of AI integration articulated in the dataset. It is also the model most consistent with emerging international frameworks for AI in medication safety, which position AI as a computational amplifier of pharmacist expertise rather than a replacement for it [19,20].

Participants’ conditional support for AI was therefore accompanied by clear distinctions between appropriate and inappropriate uses of AI. They were more accepting of AI in structured, supportive, and low-risk tasks, such as screening, flagging potential risks, or organising clinical information. However, they were more cautious when AI was applied to complex decisions requiring clinical judgement, patient interaction, procedural expertise, or professional accountability.

### 3.5. Theme 5: Experience, Specialty, and Digital Literacy as Determinants of AI Acceptance

Variation in AI acceptance was associated with three interrelated participant char-acteristics: years of clinical experience, specialty context, and self-assessed digital health literacy. These factors did not operate independently but functioned as an interlocking triad that shaped both the content and the emotional valence of participants’ orientations toward AI.

Years of clinical experience exerted a complex, non-linear influence on AI acceptance. Contrary to a simplistic linear hypothesis in which more experienced clinicians would uniformly resist AI as a threat to professional authority, the data revealed a more nuanced pattern. While highly experienced senior consultants expressed the most elaborated critiques of AI limitations, many also demonstrated the most sophisticated understanding of potential AI applications, suggesting that depth of clinical experience confers both the capacity to identify AI’s failings and the expertise to deploy it judiciously. Mid-career clinicians (10–20 years’ experience) expressed the most ambivalent positions, torn between awareness of AI’s potential efficiency gains and concern about premature reliance. Junior clinicians consistently reported the highest baseline enthusiasm but also the greatest anxiety about the absence of training and guidance.

P17, a senior endocrinologist with 22 years of practice, encapsulated the experienced clinician’s position:

*“I am not against AI—I am against the naive idea that AI can replace judgement that takes decades to develop. Use it as a tool, by all means. But give it to someone who knows enough to know when it is wrong. If a junior clinician uses AI without training, they may not know when the recommendation is unsafe.”*

Clinical specialty context shaped AI acceptance in ways that tracked the degree of quantitative data dependence, diagnostic standardisation, and regulatory exposure characteristic of different specialties. Radiologists, pathologists, and cardiologists, whose practice already involves high volumes of pattern-recognition tasks amenable to algorithmic analysis, expressed the highest conditional acceptance of AI, often with direct reference to published evidence on AI performance in their domains. P4, a radiologist, noted:

*“I have read the literature on AI in chest CT interpretation. Some of these systems genuinely perform at specialist level for specific findings. I would use them—with my eyes open and my hand still on the report.”*

By contrast, clinicians in specialties characterised by high relational and narrative content such as psychiatry, general practice, paediatrics, expressed considerably more scepticism, grounding their reservations in the irreducibility of contextual, biographical, and relational knowing to algorithmic representation. P9 reflected:

*“Psychiatry is the relationship. If you algorithmise the relationship, you have lost what heals. A checklist does not repair a therapeutic alliance.”*

Digital literacy emerged as the third critical axis. Participants who self-reported higher familiarity with electronic health records, clinical informatics platforms, or research-grade AI tools expressed notably more nuanced and differentiated views of AI capability and limitation. Those with limited digital health literacy tended toward either categorical acceptance or categorical rejection positions that lacked the discriminatory capacity of more digitally literate participants. P12, a general practitioner with limited exposure to digital health systems, reflected honestly:

*“I honestly do not know what AI in the clinic looks like. I have heard about it. But I have never seen it. So I cannot tell you if I trust it—I do not know what I would be trusting.”*

This observation foregrounds the importance of digital health literacy as a prerequisite for informed professional engagement with AI, a finding with direct implications for pre-service and continuing professional education curricula.

### 3.6. Theme 6: Jordanian Health-System Readiness: Local Guidelines, Infrastructure, and Regulation

A distinctive aspect of participants’ accounts concerned the contextual features of the Jordanian health system that they viewed as either enabling or limiting AI integration. These factors were not peripheral to their perceptions; rather, they were described as central to whether AI could be implemented safely and equitably in Jordanian clinical practice. Three sub-themes emerged: the absence of Jordan-specific clinical AI guidelines and validation frameworks, infrastructural deficiencies, and regulatory uncertainty.

#### 3.6.1. Absence of Local Clinical AI Guidelines

Participants expressed consistent concern that AI tools deployed or under consideration in Jordan had not been validated against Jordanian clinical populations, disease prevalence patterns, or prescribing norms. The absence of Jordan-specific (or even Arab-region-specific) clinical AI guidelines was experienced not merely as an inconvenience but as a foundational legitimacy deficit. P21 articulated this concern:

*“Every guideline I follow in my practice has been adapted for Jordan, or at least reviewed by Jordanian specialists. I do not apply American dosing guidelines to Jordanian patients without review. Why would AI be different?”*

This demand for local validation reflects a sophisticated understanding of the context-specificity of clinical knowledge and the risks of uncritical technology transfer from high-income to lower-income settings.

#### 3.6.2. Infrastructural Deficiencies

Participants at public sector institutions in particular described infrastructural conditions that rendered AI implementation aspirationally premature: unreliable internet connectivity, absence of interoperable electronic health record systems, insufficient hardware, and lack of institutional IT support. P16, a nurse in a public hospital in Irbid, observed:

*“We still have wards where we write on paper. There is no computer at every nursing station. I am not sure how AI enters a hospital that does not have basic digital infrastructure. It would be like building the roof before the walls.”*

The contrast between public and private sector institutional readiness was stark and frequently commented upon. Private hospital clinicians described considerably more advanced digital infrastructure, including functioning EHR systems and institutional appetite for piloting digital health tools. This sectoral divide raises equity concerns: if AI adoption proceeds first in private healthcare settings serving higher-income populations, it risks widening existing health disparities rather than ameliorating them.

#### 3.6.3. Regulatory Vacuum

Participants were unanimous in identifying the absence of dedicated AI healthcare regulation in Jordan as a critical governance gap. No participant was aware of Jordanian-specific legislation governing the use of AI in clinical contexts, the approval processes for clinical AI tools, data protection standards applicable to AI systems, or liability frameworks for AI-related adverse events. P9 expressed the collective frustration:

*“In Jordan, there is no law that tells me what AI I can use, who approves it, who is responsible when it goes wrong. There are no clear rules yet, so using AI in practice feels uncertain. The responsible thing is to be very cautious.”*

Several participants drew explicit comparisons to the regulatory frameworks governing drug approval—noting that no medication could be administered to patients without regulatory approval, and that AI clinical decision-support tools should be subject to equivalent, or at least analogous, regulatory scrutiny and post-market surveillance.

### 3.7. Theme 7: Privacy, Confidentiality, and Ethical Governance

While Theme 6 focused on organisational and regulatory readiness, Theme 7 cap-tured participants’ ethical concerns regarding how patient data would be used, stored, shared, and protected in AI-supported care. Data privacy and patient confidentiality concerns constituted a significant thematic thread across the dataset, reflecting both professional ethical commitments and pragmatic concerns about the technical and in-stitutional conditions under which patient data would be processed by AI systems. Par-ticipants’ accounts in this domain reflected awareness of both the specific privacy vul-nerabilities introduced by AI and the broader ethical governance challenges of operating AI in data-scarce and institutionally under-regulated health system contexts.

The prospect of patient data being transmitted to, stored by, or processed through third-party AI platforms, particularly those hosted on servers outside Jordan, generated substantial concern. P3, a clinical pharmacist, expressed this with specificity:

*“Medication histories, diagnoses, genomic data—these are among the most sensitive things a patient entrusts us with. If an AI system sends that data to a server in another country, does the patient know? Did they consent? Do I even know where the data goes? These are not hypothetical concerns. These are things happening now.”*

This concern reflects a sophisticated awareness of the data governance implications of cloud-based AI systems, a particularly acute consideration given Jordan’s nascent personal data protection legislative environment. Participants noted that without clearly articulated data governance frameworks specifying data minimisation principles, storage limitations, patient consent procedures, and cross-border data transfer restrictions, the ethical deployment of AI clinical tools in Jordan is fundamentally compromised.

Patient consent emerged as a specific sub-concern. Several participants questioned whether patients were being adequately informed that AI tools were being used in their clinical care. P19, an obstetrician, raised this in the context of AI-assisted foetal monitoring:

*“If AI is analysing my patient’s CTG, she should know that. She should have the right to ask how it works, who else sees the output, what it means for her care. If AI is used in patient care, patients should know how their information is being used and what it means for their care.”*

More broadly, participants identified a tension between the data-intensive requirements of AI systems—which typically require large volumes of patient data to function effectively—and the ethical principle of data minimization that underpins professional obligations of confidentiality. Several called explicitly for the development of AI-specific patient consent frameworks, distinct from existing clinical consent procedures, that adequately address algorithmic data processing.

Algorithmic bias was raised by a subset of participants as an ethical governance concern with direct equity implications. P21 reflected on the potential for AI trained on non-representative datasets to produce systematically inequitable clinical recommendations:

*“If the AI was trained mostly on data from white, male, middle-aged patients, which a lot of AI in medicine was, and I am using it for a Jordanian woman with different risk factors, different genetics, different cultural practices, I could be giving her worse care than if I had just relied on my own clinical judgement. That is not a trivial concern.”*

This articulation of algorithmic bias as an equity concern, with direct implications for the clinical care of patients who are underrepresented in training datasets, reflects an ethically sophisticated engagement with the social justice dimensions of AI healthcare governance.

### 3.8. Theme 8: Training Requirements for Safe and Responsible AI Use

The final theme concerned the training requirements participants regarded as preconditions for safe and responsible AI integration. Across professional groups, par-ticipants viewed AI literacy as a patient-safety requirement rather than an optional professional-development activity. Across all professional groups, seniority levels, and institutional contexts, there was consensus that the current workforce was substantively underprepared for the demands of AI-assisted clinical practice and that this unprepar-edness itself constituted a patient safety risk.

Training needs were articulated at multiple levels: foundational AI literacy, critical appraisal of AI outputs, technical interface proficiency, and ethical and medico-legal education. Critically, participants did not conceive of these as discrete, siloed competencies, but as integrated components of a coherent AI professional development curriculum that would need to be embedded within both pre-service education and continuing professional development frameworks.

P15 articulated the foundational literacy requirement:

*“I do not need to understand the code behind an algorithm. But I need to understand what kind of data it was trained on, what it can and cannot do, and how to critically evaluate its outputs. That is the minimum. Without that, clini-cians may use AI without understanding its limitations.”*

The critical appraisal dimension of training was emphasised by participants who drew parallels to established evidence-based medicine (EBM) competencies. Just as clinicians are trained to critically evaluate clinical research literature, they argued, AI literacy requires specific competencies in evaluating the quality, validity, and contextual appropriateness of AI tools and their outputs. P4 noted:

*“We teach residents how to read a randomised controlled trial. We need to teach them how to read an AI validation study. What was the test population? What were the outcome measures? What were the failure modes? These are not optional questions.”*

Nursing and allied health participants raised important equity considerations in relation to training access. P8, a clinical nutritionist, expressed concern that formal AI training was more likely to be directed at physicians—reinforcing professional hierarchies and leaving nursing and allied health staff operationally exposed to AI tools they lacked the competence to critically evaluate:

*“If only the doctors get trained on AI, but the nurses and allied health professionals are the ones actually interfacing with it at the bedside, that is a problem. Patient safety does not respect professional hierarchy.”*

Participants also identified the importance of simulation-based training as a modality well-suited to developing AI critical appraisal competencies in safe, low-stakes environments. Several drew on analogies to clinical skills simulation noting that practising with AI in simulated case scenarios would allow clinicians to develop familiarity, identify failure modes, and build confidence before encountering AI-assisted decision-making in high-stakes real-world contexts.

Finally, a recurring call emerged for national-level coordination of AI training for healthcare professionals in Jordan. Rather than ad hoc, institution-specific approaches, participants advocated for a nationally standardised AI competency framework analogous to national medical licensing standards that would ensure consistency of preparation across public and private sectors, professional disciplines, and geographical regions. P7 summarised this aspiration:

*“Jordan should not be waiting for each hospital to figure out AI training on its own. We need a national curriculum, national standards, national oversight. Otherwise we will have some hospitals doing it well and some doing it dangerously, and the patients will pay the price.”*

## 4. Discussion

### 4.1. Conditional Trust, Institutional Legitimacy, and Human Oversight

This study advances current understanding of AI-assisted clinical decision-making in Jordan by showing that healthcare professionals’ acceptance of AI is not simply a matter of technological enthusiasm or resistance. Rather, acceptance was constructed conditionally through the interaction of perceived technical reliability, professional accountability, institutional legitimacy, and health-system readiness. This finding moves beyond general measures of AI acceptability by showing how healthcare professionals reason through the practical conditions under which AI outputs can be trusted, questioned, documented, overridden, or integrated into clinical workflows. From a sociotechnical perspective, AI was not perceived as an isolated digital tool, but as an intervention whose safety depends on the surrounding clinical, legal, organisational, and professional systems in which it is used [12,13].

The findings also align with concepts of trust in automation and institutional trust. Participants did not describe trust as confidence in algorithmic accuracy alone. Instead, trust depended on whether AI systems were locally validated, explainable, institutionally endorsed, and embedded within clear accountability structures [6,7]. This suggests that, in the Jordanian context, trust in AI may be mediated through trust in hospitals, regulators, professional bodies, and implementation processes. For nurses, allied health professionals, and junior clinicians in particular, institutional endorsement appeared to function as a practical substitute for independent technical evaluation of AI systems.

These findings also resonate with broader digital health literature beyond AI-specific implementation. Mixed-methods work on clinician use of telemedicine has shown that engagement with digital health tools is shaped by workflow fit, user experience, institutional support, and perceived disruption to established clinical routines [29]. This is relevant to the present study because participants’ acceptance of AI was not based only on perceived technical usefulness, but also on whether AI could be integrated into everyday clinical work without increasing uncertainty, disrupting responsibility structures, or weakening professional judgement.

The distinction participants drew between AI as assistance and AI as substitution can be understood through a human-in-the-loop and sociotechnical model of clinical decision-making [8,9]. Participants were generally willing to consider AI when it supported screening, risk flagging, information organisation, or documentation, but they resisted models in which AI appeared to replace professional judgement or redistribute responsibility without clear safeguards. This distinction is important because safe AI implementation depends not only on technical performance, but also on role design: where the AI output enters the workflow, who reviews it, how disagreement is documented, when escalation is required, and who remains accountable for the final decision.

### 4.2. Accountability and AI Lifecycle Governance

The accountability concerns raised by participants demonstrate that AI implementation is not only a technical matter but also a medico-legal and organisational challenge. Participants were uncertain about who would be responsible if an AI-influenced decision resulted in patient harm: the clinician who accepted the recommendation, the hospital that approved the tool, the developer who designed it, the regulatory body that licensed it, or the wider system that allowed its use. This reflects the accountability gap described in the international AI ethics literature, where responsibility becomes difficult to assign when clinical decisions are shaped by both human and algorithmic inputs [5,30]. In this study, however, accountability was linked to real clinical hierarchies, prescribing authority, interprofessional communication, and legal exposure.

The accountability concern identified in this study may also be understood as a form of distributed or diffused responsibility within AI-assisted clinical decision-making [31,32]. Participants did not view responsibility as disappearing when AI entered the workflow; rather, they perceived responsibility as becoming harder to locate because decision-making was distributed across clinicians, institutions, software developers, data sources, and regulatory bodies. This explains why AI could simultaneously be perceived as a support tool and as a medico-legal risk. In sociotechnical terms, the problem is not only who is formally liable after harm occurs, but whether the system provides clinicians with sufficient authority, transparency, documentation pathways, and escalation mechanisms to meaningfully evaluate and contest AI outputs before harm occurs.

Participants’ emphasis on local validation and system readiness also points to the importance of lifecycle governance for AI-enabled healthcare systems. AI safety does not end once a tool is approved or introduced into a hospital workflow. Healthcare organizations need procedures for pre-deployment validation, controlled piloting, user training, documentation of AI-assisted decisions, monitoring of overrides, reporting of AI-related incidents, periodic revalidation, and detection of performance drift over time [31,33]. In practice, this requires a broader implementation-governance approach in which AI tools are not only validated before deployment, but also monitored after implementation for performance, data quality, bias, usability, workflow fit, user training needs, and clinical impact. This broader framing is consistent with recent literature highlighting that successful AI integration in healthcare depends on organisational readiness, governance, staff preparation, implementation planning, and alignment with clinical workflows [34].

Human oversight should therefore be operationalized rather than treated as a general principle. In practical terms, institutions could define which AI outputs require mandatory clinician review, which outputs require pharmacist review, how AI recommendations should be documented in the medical record, when clinicians should provide reasons for overriding AI outputs, and how concerns should be escalated to supervisors, medication-safety committees, or digital health governance teams. This type of workflow-level oversight is particularly important in high-risk domains such as prescribing, diagnostic triage, and deterioration prediction, where AI recommendations may influence time-sensitive clinical decisions.

### 4.3. Medication-Related Safety as an Exploratory Cross-Cutting Concern

Medication-related safety emerged as an important but exploratory cross-cutting concern. Participants who discussed prescribing and therapeutic decision-making viewed AI-supported medication recommendations as potentially useful but clinically high risk, particularly when applied to dosing, drug-drug interactions, drug-herb interactions, renal or hepatic adjustment, polypharmacy, and therapeutic monitoring. These concerns are consistent with evidence from clinical decision-support systems showing that digital tools may improve medication safety when well designed and integrated into workflows, but may also introduce new risks when poorly calibrated, poorly contextualized, or over-relied upon [19,20,35]. However, because pharmacists represented a small subgroup of the sample, these findings should not be interpreted as a comprehensive account of pharmacists’ perspectives. Rather, they suggest that medication-related AI tools require further focused investigation, particularly regarding how pharmacists, prescribers, and medication-safety committees can contribute to validation, implementation, and oversight.

### 4.4. Health-System Readiness and Transferability to LMIC Settings

Health-system readiness was another major determinant of participants’ views. AI was not perceived as a stand-alone technology that could simply be added to existing clinical practice. Participants identified several prerequisites for safe implementation, including reliable digital infrastructure, interoperable electronic health records, technical support, Arabic-language usability, local validation, regulatory approval pathways, data governance, and workforce training. These findings are consistent with the wider literature on digital health implementation in LMICs, where infrastructure, governance capacity, organizational readiness, and workforce preparedness are often decisive determinants of whether technologies improve care or deepen existing weaknesses [10,11].

Ethical governance was closely linked to this readiness agenda. Participants raised concerns about privacy, confidentiality, patient consent, cross-border data transfer, and algorithmic bias. These concerns are particularly relevant because AI systems often require access to large volumes of sensitive clinical data. In practical terms, existing confidentiality procedures may not be sufficient for AI-assisted care. Healthcare institutions may need specific policies that clarify when AI is used, what data are processed, who has access to outputs, how recommendations are documented, and how patients are informed in a clear and culturally appropriate manner [30,36].

Although this study was conducted in Jordan, several findings are likely transferable to other LMIC settings where AI implementation is shaped by uneven digital infrastructure, limited local validation capacity, evolving regulatory frameworks, workforce training gaps, and dependence on externally developed AI tools. The emphasis on conditional trust, human oversight, data governance, and post-deployment monitoring is therefore likely to have broader relevance beyond Jordan. However, some findings may be more context-specific, including concerns related to Jordanian prescribing practices, Arabic-language usability, local clinical workflows, public-private sector differences, and the current state of national AI regulation. Readers should therefore assess transferability in relation to the similarity of their own healthcare systems, regulatory environments, and digital health maturity.

### 4.5. Practical Implications for Stakeholders

The practical implications of these findings are summarised in Table 4. These implications should be interpreted as practice- and policy-relevant directions derived from participants’ accounts, rather than as definitive policy prescriptions.

Overall, the findings suggest that responsible AI integration in Jordanian healthcare will require more than technological availability. Participants’ accounts indicate that safe adoption depends on institutional credibility, local validation, regulatory clarity, ethical data practices, workforce preparation, and workflows that preserve professional judgement. Their cautious but constructive stance should not be interpreted as resistance to innovation. Rather, it reflects a patient-safety-oriented view of AI adoption in which digital tools must earn professional trust through evidence, accountability, and fit with local clinical realities.

## 5. Limitations

Several limitations should be acknowledged. First, the study was conducted in three of Jordan’s twelve governorates, mainly including major urban and semi-urban healthcare settings. Although Amman, Irbid, and Zarqa include important public, private, and university-affiliated healthcare institutions, the findings may not fully reflect the views of healthcare professionals working in rural, remote, or under-resourced facilities where digital infrastructure, staffing patterns, and institutional readiness for AI may differ substantially. Therefore, transferability to other Jordanian settings should be assessed with attention to local healthcare resources, digital maturity, and organizational context.

Second, the sample was purposively selected and included healthcare professionals with at least some awareness of digital health or AI-related tools. This approach was appropriate for the study aim, but it may have introduced selection bias toward participants who were more interested in or more reflective about AI than the wider healthcare workforce. In addition, physicians represented half of the sample, while pharmacists and allied health professionals were rep-resented by only three participants each. Because medication-related safety and prescribing concerns emerged as cross-cutting findings, the limited number of pharmacists should be considered when interpreting these results. The pharmacotherapy-related findings should therefore be viewed as exploratory and not as a comprehensive representation of pharmacists’ perspectives. A larger pharmacist sample may have generated deeper or different insights into AI-assisted prescribing, medication reconciliation, dose adjustment, drug-interaction management, and therapeutic monitoring. Similarly, the small allied health subgroup means that allied health perspectives may not have been fully captured. The study also does not claim discipline-specific saturation within these small subgroups; saturation was assessed at the overall dataset level.

Third, the study examined healthcare professionals’ perceptions of AI-assisted clinical decision-making rather than directly observing AI use in routine clinical workflows. Participants varied in their level of exposure to AI and digital decision-support systems; some reflected on direct or indirect experience with AI-enabled or algorithm-supported tools, while others discussed anticipated or hypothetical future applications. As a result, the findings should be interpreted as professional perceptions, expectations, and readiness-related concerns rather than evidence of actual AI-use behaviour in Jordanian clinical practice.

Fourth, social desirability bias may have influenced participants’ accounts. Because AI is a highly visible and professionally important topic, some participants may have presented themselves as more open to innovation, more cautious about patient safety, or more aware of ethical and governance issues than they would be in routine practice. To reduce this risk, interviews were conducted confidentially, participants were reminded that there were no right or wrong answers, and the analysis considered both supportive and sceptical views. Nevertheless, social desirability bias cannot be excluded.

Fifth, most interviews were conducted in Arabic and translated into English for analysis and reporting. Although translation, back-translation of a subsample, and team discussion were used to support semantic accuracy, some loss of nuance is unavoidable when translating professional and culturally situated expressions. Quotations were edited only minimally for grammar, readability, and removal of identifying details; however, translated quotations may not fully capture the tone, register, or idiomatic features of the original Arabic accounts.

Sixth, as with all reflexive qualitative research, the findings reflect an interpretive analytic process shaped by participants’ accounts, researcher reflexivity, and the disciplinary backgrounds of the research team. Although reflexive memos, peer debriefing, audit trails, and team discussions were used to enhance transparency, the researchers’ expertise in clinical pharmacy, pharmacotherapy, health informatics, and health-professions education may have influenced the interpretation of medication-safety, accountability, and implementation-readiness themes. This risk was addressed by reviewing medication-related codes separately, avoiding construction of pharmacotherapy as an independent theme, and reporting medication-related safety as a cross-cutting concern rather than a pharmacist-specific conclusion.

Finally, AI technologies, regulatory discussions, and healthcare implementation contexts are evolving rapidly. Participants’ perceptions may therefore change as AI tools become more familiar, more locally validated, more widely regulated, or more embedded in clinical workflows. Future research should include larger and more profession-specific samples, particularly pharmacists and nurses, and should examine actual AI implementation in clinical settings using observational, mixed-methods, or longitudinal designs.

## 6. Conclusions

This study provides qualitative insight into healthcare professionals’ perceptions of AI-assisted clinical decision-making in Jordan. Participants expressed cautious but constructive views, recognizing the potential value of AI while emphasising that its safe use depends on local validation, transparent governance, clear accountability, ethical data practices, workforce training, and preservation of professional judgement. AI was viewed as a support tool rather than a replacement for clinical decision-making. Medication-related safety emerged as an important cross-cutting concern, suggesting that AI recommendations affecting prescribing, dosing, interactions, or therapeutic monitoring require careful professional review.

Overall, the findings suggest that responsible AI implementation in Jordanian healthcare should be guided not only by technological capability, but also by patient safety, professional accountability, institutional readiness, and contextual fit. Context-sensitive governance strategies, interprofessional training, equitable digital infrastructure, and clearly defined human oversight mechanisms may help support trustworthy and professionally accountable AI adoption.

## Figures and Tables

**Table 1:** Participant Characteristics (*n* = 22).

Characteristic	Category	*n* (%)
Professional role	Physician	11 (50.0)
	Nurse	5 (22.7)
	Pharmacist	3 (13.6)
	Allied HP	3 (13.6)
Institution type	Private Hospital	8 (36.4)
	General Hospital	6 (27.3)
	University Hospital	4 (18.2)
	Primary Health Centre	3 (13.6)
	Rehabilitation Centre	1 (4.5)
Seniority	Junior	4 (18.2)
	Mid-Level	6 (27.3)
	Senior	12 (54.5)
Governorate	Amman	14 (63.6)
	Irbid	5 (22.7)
	Zarqa	3 (13.6)
Gender	Male	11 (50.0)
	Female	11 (50.0)

Note. HP = Health Professional. Percentages were calculated using the total sample size (*n* = 22).

**Table 2:** Summary of Themes: Analytical Focus and Representative Quotations.

Theme	Analytical Focus	Representative Quotation
Conditional Trust in AI-Assisted Clinical Decision-Making	AI was treated as a provisional, institutionally mediated support tool rather than an independent decision-maker.	“*I would not fully trust it immediately. It needs to prove that it works safely in our patients and in our hospital.*” (P7, Senior Cardiologist)
Accuracy, Evidence, and the Risk of Clinical Misinformation	Participants were concerned about confident but incorrect outputs, especially when AI recommendations affected diagnosis or prescribing.	“*The problem is that AI may give an answer with confidence. If the answer is wrong, a junior doctor may not feel able to question it.*” (P11, Consultant Surgeon)
Accountability, Liability, and Professional Responsibility	AI complicated responsibility for clinical decisions, particularly when prescribing recommendations contributed to patient harm.	“*There is no clear answer to that question in Jordanian law.*” (P9, Senior Psychiatrist)
AI as an Adjunct to, Not a Substitute for, Clinical Judgment	AI was acceptable for screening and support tasks, while final clinical and therapeutic reasoning remained human responsibilities.	“*AI may read a score, but we also look at the patient’s face, breathing, pain, and general condition*.” (P6, Senior Oncology Nurse)
Experience, Specialty, and Digital Literacy as Determinants of AI Acceptance	Acceptance varied by seniority, specialty, and digital literacy, with junior clinicians more enthusiastic but potentially more vulnerable to over-reliance.	“*If a junior clinician uses AI without training, they may not know when the recommendation is unsafe.*” (P17, Senior Endocrinologist)
Jordanian Health-System Readiness	Safe implementation was constrained by limited local validation, Arabic-language barriers, infrastructure gaps, and regulatory uncertainty.	“*There are no clear rules yet, so using AI in practice feels uncertain.*” (P9, Senior Psychiatrist)
Privacy, Confidentiality, and Ethical Governance	Participants emphasized consent, data security, cross-border data transfer, and algorithmic bias as core governance concerns.	“*If AI is used in patient care, patients should know how their information is being used.*” (P19, Senior Obstetrician)
Training Requirements for Safe and Responsible AI Use	Participants called for national training in AI literacy, critical appraisal, and safe integration into clinical workflows.	“*Without training, clinicians may use AI without understanding its limitations.*” (P15, Consultant Neurologist)

Note: Italics are used in the “Representative quotation” column to distinguish verbatim participant quotations from the authors’ analytical interpretation.

**Table 3:** Variation in AI-related perceptions across professional groups.

Professional Group	Main Concerns Raised	Distinctive Emphasis
Physicians	Diagnostic accuracy, clinical autonomy, liability, conflict between AI output and clinical judgement	AI may support diagnosis, triage, and information retrieval, but final responsibility remains with the physician of record.
Pharmacists	Dosing, drug-drug interactions, drug-herb interactions, renal adjustment, polypharmacy, medication reconciliation	AI-assisted prescribing should be reviewed through medication-safety and pharmacotherapy expertise, especially for high-risk medicines and complex patients.
Nurses	Institutional approval, workflow clarity, escalation, bedside safety, communication hierarchy	Trust in AI was strongly mediated by hospital endorsement and clear procedures for questioning or escalating AI-supported decisions.
Allied health professionals	Training access, digital literacy, role clarity, inclusion in implementation	AI training and implementation should include all professionals who interact with AI-supported workflows, not only physicians.

**Table 4:** Findings, implementation implications, and stakeholders.

Finding	Implementation Implication	Main Stakeholders
Trust in AI was conditional	AI tools should undergo transparent local validation before routine clinical use.	Hospitals, regulators, professional bodies, AI developers
Participants feared confident but incorrect outputs	Training should address automation bias, critical appraisal of AI outputs, and recognition of AI failure modes.	Universities, CPD providers, clinicians, educators
Accountability was unclear	Institutions should define responsibility for accepting, challenging, documenting, or overriding AI recommendations.	Hospitals, legal authorities, regulators, clinicians
AI was accepted as support, not substitution	AI should be implemented as human-supervised decision support rather than autonomous decision-making.	Hospital leadership, clinicians, AI developers
Medication-related safety was a cross-cutting concern	AI tools affecting prescribing, dosing, interactions, or monitoring should involve medication-safety expertise and, where appropriate, pharmacist review.	Pharmacists, prescribers, medication-safety committees
System readiness was uneven	AI implementation should consider infrastructure, EHR interoperability, Arabic-language usability, and public-sector capacity.	Ministry of Health, hospitals, IT departments
Privacy and bias were major concerns	AI governance should address consent, data minimisation, cross-border data transfer, bias detection, and auditability.	Regulators, ethics committees, hospitals, AI vendors
Workforce training was viewed as essential	Interprofessional AI literacy training should be introduced before routine implementation.	Universities, professional councils, hospitals

## Data Availability

Due to the qualitative nature of the study and the need to protect participant confidentiality, full interview transcripts and potentially identifiable data are not publicly available.

## Supplementary Materials

The following supporting information can be downloaded at: https://www.mdpi.com/article/10.3390/healthcare14121724/s1, Supplementary File S1: Completed COREQ checklist; Supplementary File S2: Semi-structured interview guide; Supplementary File S3: Coding tree illustrating the progression from selected initial codes to candidate themes and final themes.

## Abbreviations

The following abbreviations are used in this manuscript:

AI	Artificial intelligence
CDSS	Clinical decision-support system
COREQ	Consolidated Criteria for Reporting Qualitative Research
EHR	Electronic health record
IRB	Institutional Review Board
LMIC	Low- and middle-income country
