# Supplementary material for: Healthcare Professionals’ Perceptions of AI-Assisted Clinical Decision-Making in Jordan: A Qualitative Study of Trust, Accountability, System Readiness, and Professional Practice
Source: Healthcare (Basel). 2026 Jun 15;14(12):1724. doi: 10.3390/healthcare14121724 (PMC13300382; doi:10.3390/healthcare14121724)
Supplement: Supplementary file 1 [file healthcare-14-01724-s001.zip › Supplementary Material S1-COREQ checklist.pdf]

Supplementary Material S1: COREQ 32-Item Checklist

No.	Item	Sub-item	Reported Response
**Domain 1: Research Team & Reflexivity**			
1	Interviewer/facilitator	Characteristics	PI: clinical pharmacy and health informatics background; doctoral-level qualitative training. Section 2.1.1.
2	Credentials	Academic qualifications	PhD/MD-level; university-affiliated team. Section 2.1.1.
3	Occupation	Employment	Academic researchers; no clinical employment at study sites. Section 2.1.1.
4	Gender	Gender	Mixed-gender research team. Section 2.1.1.
5	Experience & training	Qualitative experience	PI: 8+ years qualitative research; trained co-coder. Section 2.1.1.
6	Relationship with participants	Prior relationship	None prior; first contact via institutional gatekeepers. Section 2.1.2.
7	Participant knowledge of interviewer	Knowledge of interviewer	Academic affiliation and study aims disclosed at outset. Section 2.1.2.
8	Interviewer characteristics	Characteristics disclosed	Professional background disclosed; reflexive journals maintained. Section 2.1.3.
**Domain 2: Study Design**			
9	Methodological orientation & theory	Framework	Reflexive Thematic Analysis (Braun & Clarke, 2006; 2021); constructivist

No.	Item	Sub-item	Reported Response
			epistemology; phenomenological orientation. Section 2.2.1.
10	Sampling	Method	Purposive; maximum variation — discipline, specialty, seniority, gender, sector, location; deliberate pharmacist representation. Section 2.2.2.
11	Method of approach	Recruitment method	Institutional gatekeeper introduction; written invitation; follow-up. Section 2.2.2.
12	Sample size	Number	n=22; thematic saturation confirmed at n=20. Section 2.2.5.
13	Non-participation	Refusals/dropouts	9 non-participants: 5 declined, 4 non-responsive. Section 2.2.6.
14	Setting	Location	Private rooms; 8 sites; Amman, Irbid, Zarqa; November 2025 – February 2026. Section 2.2.3.
15	Presence of non-participants	Others present	None. Section 2.2.3.
16	Description of sample	Sample description	See Table 1. Section 2.2.2.
17	Interview guide	Questions/topics	Semi-structured; pilot-tested with clinical informatics and pharmacy specialists; covers trust, accuracy, prescribing accountability, medication safety, training, ethics. Full interview guide

No.	Item	Sub-item	Reported Response
			provided as Supplementary Appendix B. Section 2.2.4.
18	Repeat interviews	Conducted	Not conducted. Section 2.2.4.
19	Audio/visual recording	Use of recording	Audio-recorded with written consent. Section 2.2.4.
20	Field notes	Notes taken	Reflexive field notes immediately post-interview. Section 2.2.4.
21	Duration	Interview duration	45–90 minutes (mean 62 minutes); data collection November 2025 – February 2026. Section 2.2.3 and 2.2.4.
22	Data saturation	Saturation	Interim analysis every 5 interviews; saturation at n=20. Section 2.2.5.
23	Transcripts returned	Member checking	Theme summaries shared with 8-participant subsample; feedback incorporated. Section 2.3.3.
**Domain 3: Analysis & Findings**			
24	Number of data coders	Coders	Two independent coders; disagreements resolved by discussion. Section 2.3.1.
25	Description of coding tree	Tree	Inductive; NVivo 14; RTA six phases. Section 2.3.1.
26	Derivation of themes	Inductive/deductive	Primarily inductive; connections to accountability theory and medication safety frameworks noted analytically. Section 2.3.2.

No.	Item	Sub-item	Reported Response
27	Software	Analysis tool	NVivo 14 (QSR International). Section 2.3.1.
28	Participant checking	Member checking	8-participant subsample; refinements incorporated. Section 2.3.3.
29	Quotations presented	Sufficient quotations	Representative quotations; participant IDs used. Section 3 and Table 2.
30	Consistency between data & findings	Coherence	Audit trail; peer debriefing; reflexive memos. Section 2.3.1.
31	Clarity of major themes	Themes clear	8 themes with labels, definitions, and illustrative data. Section 3 and Table 2.
32	Clarity of minor themes	Minor themes	Sub-themes in Themes 5 and 6 reported separately. Sections 3.5 and 3.6.
