# Supplementary material for: Healthcare Professionals’ Perceptions of AI-Assisted Clinical Decision-Making in Jordan: A Qualitative Study of Trust, Accountability, System Readiness, and Professional Practice
Source: Healthcare (Basel). 2026 Jun 15;14(12):1724. doi: 10.3390/healthcare14121724 (PMC13300382; doi:10.3390/healthcare14121724)
Supplement: Supplementary file 1 [file healthcare-14-01724-s001.zip › Supplementary Material S2-Interview Guide.pdf]

Supplementary Material S2

Semi-Structured Interview Guide

Study title: Healthcare Professionals' Perceptions of AI-Assisted Clinical Decision-Making in Jordan: A Qualitative Study of Trust, Accountability, System Readiness, and Professional Practice

Note: This semi-structured guide was used flexibly. Questions were not necessarily asked verbatim or in the same order in every interview. Probes were used depending on the participant's professional role, clinical experience, and responses.

Opening Script

Thank you for agreeing to participate in this interview. The purpose of this study is to explore healthcare professionals' perceptions of artificial intelligence (AI)-assisted clinical decision-making in Jordan, including issues related to trust, accuracy, accountability, prescribing safety, pharmacotherapy, privacy, system readiness, and training needs. Participation is voluntary, and you may decline to answer any question or withdraw at any time. With your permission, the interview will be audio-recorded for transcription and analysis. Your identity and institution will remain confidential, and no identifying information will be reported.

Participant Background

Before starting the main interview questions, the interviewer recorded basic professional characteristics, including professional role, clinical specialty or practice area, years of experience, sector of practice, institution type, governorate, gender if voluntarily disclosed, and previous exposure to AI or digital health tools.

Interview Domains and Questions

Domain	Core interview questions	Suggested prompts/probes
**1. Professional role and clinical decision-making**	1. Can you briefly describe your current clinical role and your usual involvement in clinical decision-making?<br>2. What types of clinical decisions do you commonly make or contribute to in your daily practice?	Clarify prescribing authority, medication-related responsibilities, interprofessional collaboration, and whether the participant works in acute care, outpatient care, community pharmacy, or another setting.
**2. Current exposure to digital tools and AI**	3. What digital tools, electronic systems, or clinical decision-support tools do you currently use in your practice?<br>4. When you hear the term "AI in healthcare," what comes to mind?	Explore electronic health records, drug information databases, clinical calculators, imaging tools, decision-support systems, generative AI, and whether AI is used formally or informally.
**3. Trust in AI-assisted clinical decision-making**	5. Under what conditions would you trust AI to support a clinical decision?<br>6. Can you describe a situation in which you would accept, question, or reject an AI-generated recommendation?	Probe institutional approval, transparency, explainability, evidence base, clinician experience, specialty context, and whether AI confirms or conflicts with clinical judgment.
**4. Accuracy, evidence, and clinical misinformation**	7. What concerns do you have about the accuracy of AI-generated clinical recommendations?<br>8. How would you verify whether an AI recommendation is clinically appropriate?	Probe outdated information, hallucinated or unsupported recommendations, guideline alignment, local applicability, confident but incorrect outputs,

	9. Are there clinical areas where AI seems safer or riskier to use?	diagnosis versus treatment, and high-risk clinical decisions.
**5. Pharmacotherapy and prescribing safety**	10. How do you perceive the use of AI in prescribing or medication-related decisions?<br>11. What medication-related tasks would you be comfortable using AI for?<br>12. What medication-related tasks would you not delegate to AI?	Probe drug selection, dosing, renal or hepatic adjustment, drug interactions, drug-herb interactions, polypharmacy, antimicrobial prescribing, high-risk medications, local formularies, and medication reconciliation.
**6. Accountability and professional responsibility**	13. If an AI-influenced decision leads to patient harm, who should be responsible?<br>14. How should clinicians document the use of AI in clinical decision-making?<br>15. How might AI affect professional autonomy or interprofessional communication?	Probe prescriber responsibility, pharmacist review, nursing concerns, institutional liability, software developers, legal frameworks, hierarchy, and situations where AI conflicts with clinician judgment.
**7. Jordanian health-system readiness**	16. How ready do you think the Jordanian healthcare system is for AI-assisted clinical decision-making?<br>17. What local adaptations would be necessary before AI tools could be safely used in Jordan?	Probe public versus private sector differences, electronic health records, infrastructure, Arabic-language capability, local guidelines, local validation, insurance and formularies, workforce readiness, and national regulation.
**8. Privacy, confidentiality, and ethical governance**	18. What concerns do you have about entering patient information into AI tools?<br>19. Should patients be informed when AI is used to support their care?<br>20. What ethical safeguards are needed for AI use in healthcare?	Probe data storage, third-party platforms, cross-border data transfer, consent, confidentiality, algorithmic bias, fairness, patient autonomy, and governance.
**9. Training and implementation needs**	21. What training would healthcare professionals need before using AI safely?<br>22. Who should be responsible for providing or regulating this training?<br>23. What would responsible implementation of AI in Jordanian healthcare look like?	Probe AI literacy, prompt use, critical appraisal, recognizing limitations, pharmacotherapy-specific competencies, simulation-based training, undergraduate education, continuing professional development, and national standards.
**10. Closing reflection**	24. Is there anything else you would like to add about AI-assisted clinical decision-making, prescribing safety, or the future of healthcare practice in Jordan?	Ask whether the participant wants to clarify any earlier answer or add examples from their own practice.

Interviewer Closing Statement

Thank you for sharing your views. Your responses will be anonymized and analyzed together with other interviews. If needed, we may contact you to clarify your responses or to share a summary of preliminary themes for feedback. Do you have any final questions about the study?
