# Supplementary material for: Healthcare Professionals’ Perceptions of AI-Assisted Clinical Decision-Making in Jordan: A Qualitative Study of Trust, Accountability, System Readiness, and Professional Practice
Source: Healthcare (Basel). 2026 Jun 15;14(12):1724. doi: 10.3390/healthcare14121724 (PMC13300382; doi:10.3390/healthcare14121724)
Supplement: Supplementary file 1 [file healthcare-14-01724-s001.zip › Supplementary Material S3 Coding Tree.pdf]

Supplementary Material S3. Coding Tree

This supplementary coding tree illustrates how selected initial codes were grouped into candidate themes and then refined into the final thematic structure. The examples below are illustrative rather than exhaustive. Coding was conducted at semantic and latent levels, and final themes were developed through iterative comparison of transcripts, coded excerpts, reflexive memos, and team discussions.

Selected initial codes	Candidate theme	Final theme / cross-cutting concern
AI must be tested locally; hospital approval matters; trust depends on evidence; AI should prove itself first; transparency is needed before use	Conditional trust and local validation	Theme 1. Conditional trust in AI-assisted clinical decision-making
AI may sound confident but be wrong; confidence scores can mislead; junior clinicians may over-rely; Western data may not fit Jordanian patients; AI may miss local clinical context	Accuracy, evidence, and clinical misinformation	Theme 2. Accuracy, evidence, and the risk of clinical misinformation
Who is liable if AI is wrong; clinician of record remains responsible; unclear Jordanian law; AI recommendation conflicts with clinician judgement; uncertainty about documentation and escalation	Liability uncertainty and distributed responsibility	Theme 3. Accountability, liability, and professional responsibility
AI should support not replace; final decision must remain human; useful for screening or flagging; professional judgement must be preserved; AI output requires clinical review	Human oversight and adjunctive AI use	Theme 4. AI as an adjunct to, not a substitute for, clinical judgement
Junior clinicians more enthusiastic; senior clinicians more cautious; specialty affects comfort with AI; radiology/cardiology more accepting; limited digital literacy leads to uncertainty	Professional variation in AI acceptance	Theme 5. Experience, specialty, and digital literacy as determinants of AI acceptance
No local AI guidelines; weak infrastructure; limited EHR interoperability; public-private readiness gap; no clear regulation; Arabic-language limitations	Implementation readiness and health-system constraints	Theme 6. Jordanian health-system readiness
Where does patient data go; consent for AI use; data stored outside Jordan; confidentiality concerns; algorithmic bias; non-representative training datasets	Ethical and data governance	Theme 7. Privacy, confidentiality, and ethical governance
Need AI literacy; clinicians should understand limitations; training on critical appraisal; simulation before use; national standards; interprofessional training needed	AI literacy and workforce preparation	Theme 8. Training requirements for safe and responsible AI use
Dosing risk; drug-drug interactions; drug-herb interactions; polypharmacy; renal-dose adjustment; medication reconciliation; pharmacist review when AI affects prescribing	Medication-related safety codes across several themes	Cross-cutting concern. Medication-related safety

Note. The medication-related safety codes were not treated as a stand-alone pharmacist-specific theme because the pharmacist subgroup was small. Instead, these codes were interpreted as a cross-cutting concern where they helped clarify broader issues of trust, accuracy, accountability, human oversight, and system readiness.
